# Semi-Field Evaluation and Genotoxicity of Chlorophyllin Applied Against *Aedes aegypti* Larvae (Diptera, Culicidae)

**DOI:** 10.3390/insects16030255

**Published:** 2025-03-01

**Authors:** Magda H. Rady, Asmaa M. Ammar, Areej A. Al-Khalaf, Abdelwahab Khalil, May A. Azzam, Ayman A. Abdel-Shafi, Shaimaa M. Farag

**Affiliations:** 1 Entomology Department, Faculty of Science, Ain Shams University, Abbasia, Cairo 15611, Egypt; drmagdaradi@yahoo.com (M.H.R.); shaimaa.mahmoudfarag@yahoo.com (S.M.F.); 2 Medical Parasitology Department, Faculty of Medicine, Ain Shams University, Abbasia, Cairo 1181, Egypt; 3 Biology Department, College of Science, Princess Nourah Bint Abdulrahman University, Riyadh 11671, Saudi Arabia; aaalkhalaf@pnu.edu.sa; 4 Entomology Division, Zoology Department, Faculty of Science, Beni-Suef University, Beni-Suef 62521, Egypt; abdelwahab.khalil@science.bsu.edu.eg; 5 Biochemistry Department, Faculty of Pharmacy, Cairo University, Cairo 11562, Egypt; mai.azzam@pharma.cu.edu.eg; 6 Chemistry Department, Faculty of Science, Ain Shams University, Abbasia, Cairo 11566, Egypt; aaashafi@sci.asu.edu.eg

**Keywords:** photosensitizers, bio-pesticide, larvicidal activity, genotoxicity, RAPD-PCR, histopathology, integrated pest management

## Abstract

Dengue fever is a mosquito-borne viral infection that recently appeared in Upper Egypt. Globally, more than 50 million new infections occur annually. It currently lacks effective treatment, necessitating vector control strategies targeting *Aedes aegypti*. This study investigates the potential of chlorophyllin as a control agent against dengue vectors. Chlorophyllin was characterized by FTIR analysis. The singlet oxygen quantum yield was determined by comparing the luminescence intensity at 1270 nm with that of phenalenone, yielding a value of 0.18. LC_50_ and LC_90_ values were calculated for chlorophyllin. Its larvicidal efficacy was assessed, revealing an LC_50_ of 0.47 ppm in controlled laboratories and 93.3 ppm in semi-field conditions, demonstrating its superior potency against *Aedes aegypti* compared to pheophorbide and *Bacillus sphaericus*. Genotoxicity was analyzed through Random Amplified Polymorphic DNA (RAPD)-PCR, and histopathological changes were documented through microscopic examination. The genotoxicity results revealed high similarity in the DNA configurations of chlorophyllin-treated larvae and healthy individuals (similarity index of 0.8), whereas pheophorbide and *Bacillus sphaericus* exhibited substantial genetic deviations. Histopathological analysis demonstrated severe disruptions in chlorophyllin-treated larvae’s gut epithelial cells and muscle tissues, including epithelial detachment and irregular cell shapes. These findings position chlorophyllin as a promising gut toxin larvicide for *Aedes aegypti* control, with a more favorable genetic safety profile than conventional chemicals.

## Simple Summary

Dengue fever is a serious illness transmitted by mosquitoes, affecting many people in tropical and subtropical regions. Currently, there are no specific treatments available for this disease, making it crucial to find effective ways to control the mosquito population. This study explores the use of chlorophyllin, a natural compound derived from chlorophyll, as a potential method for killing mosquito larvae. Our research showed that chlorophyllin is highly effective, with a strong ability to reduce larvae numbers in laboratory tests and in more natural settings. Additionally, we found that using chlorophyllin does not significantly alter the genetic makeup of the treated mosquito larvae compared to traditional pesticides, which can cause harmful effects. This finding suggests that chlorophyllin could be a safer alternative for controlling mosquito populations. By using chlorophyllin, we can help reduce the spread of dengue fever, contributing to better public health and safer communities.

## 1. Introduction

Mosquitoes are vectors of dangerous epidemic diseases. Traveling and global warming help in expanding vectors such as *Aedes, Anopheles*, and *Culex* spp. all over the world [1,2]. *Aedes aegypti*, the principal vector of the dengue, Zika, chikungunya, and yellow fever viruses, poses considerable public health challenges globally. The tropical and subtropical climate of Egypt creates an ideal environment for *Aedes aegypti* proliferation, particularly in urban areas, where water storage practices and inadequate drainage facilitate breeding [3]. Recent studies have confirmed the establishment of *Aedes aegypti* in various regions of Egypt, including the Red Sea Governorate [3,4]. The re-emergence of this mosquito species in Egypt has been linked to climate change, which has expanded its range and increased the risk of dengue outbreaks [5]. This necessitates the exploration of innovative and sustainable control measures.

The excessive use of chemical insecticides harms human health and the ecosystem [6], so there is an urgent need for alternative control strategies that are effective and environmentally friendly. Developing insecticides from plant sources may reduce the negative effects caused by such chemicals.

Chlorophyll derivatives, as natural porphyric compounds, have been explored as effective photosensitizing agents for controlling medical and agricultural pests [7]. A novel method has been developed using these porphyric derivatives as sunlight-activated insecticides for the outdoor control of harmful insects [8]. Photosensitizers are known to absorb various wavelengths across the solar spectrum. This allows them to undergo highly efficient photoexcitation with sunlight and generate a high quantum yield of singlet oxygen (^1^O_2_) and other reactive oxygen species (ROS) when exposed to light. These reactive species can potentially damage and kill specific stages of insects [9]. Some photo-pesticides have been identified as promising larvicides for controlling *Anopheles* spp. [10,11]. Chlorophyllin is a water-soluble derivative of chlorophyll. Compared to chlorophyll, chlorophyllin is more hydrophilic, possesses coloring capabilities, and is more stable in acidic media and light [12]. Chlorophyll derivatives, as potential larvicides, offer additional benefits such as cost-effectiveness, as well as being naturally sourced from green plants and approved by the Food and Drug Administration (FDA) as food additives (E 170) [8,9]. Chlorophyllin has not been demonstrated as sufficiently insecticidal against various arthropod vectors, and its application against *Aedes aegypti* larvae, particularly in a semi-field setting, has not been extensively studied. Furthermore, understanding the genotoxic potential of chlorophyllin is crucial for assessing its safety and suitability as an alternative larvicidal agent. This study aims to evaluate the effectiveness of chlorophyllin in controlling *Aedes aegypti* larvae under semi-field conditions, and to critically assess its genotoxic impact. By addressing these objectives, we seek to contribute to the development of environmentally safe and effective strategies for managing *Aedes aegypti* populations in Egypt, ultimately enhancing public health outcomes.

## 2. Materials and Methods

### 2.1. Pesticides Used

#### 2.1.1. Chlorophyll Derivative Preparation (Ch-L)

Chlorophyll was extracted from plant leaves (*Spinacia oleracea*; family: Amaranthaceae) and vegetable peels. A semi-synthetic combination of sodium–copper complex made from plant-extracted chlorophyll is known as chlorophyllin. In this process, the copper atom replaces the magnesium atom in the center of the chlorophyll, which no longer has a phytol tail [13,14]. Ethanol (65%) was added to the collected plant leaves before being pulverized with a mortar and pestle and heated to 60 °C in a water bath for 10 min. NaOH (20 mL-5%) was added for the saponification process, with constant stirring. The preparation was centrifuged for 10 min at 4500 rpm. After gathering the supernatant, CuSO_4_ (15 mL of 20%) solution was added. After 30 min in the water bath, Cu-chlorophyllin was extracted. Then, 2% NaOH solution was added, reaching pH 9.6, and soluble Na-Cu-chlorophyllin complexes were prepared. They were then centrifuged for 15 min at 4500 rpm. The pellets were collected, allowed to dry, and then stored in vials for further investigations [15].

#### 2.1.2. Pheophorbide

Pheophorbide was obtained from Sigma-Aldrich Chemie GmbH (Taufkirchen, Germany) and is recognized for its photodynamic activity; it has a photodynamic activity due to generating reactive oxygen species (ROS) under light. This feature is connected to its toxicity.

#### 2.1.3. *Bacillus sphaericus* (*Bs*)

A subculture of the larvicidal bacterium *B. sphaericus* was obtained from the Microbiological Resources Centre (Cairo Mircen), Faculty of Agriculture, Ain Shams University. It was kept as a slant in our lab, and different concentrations were prepared for bioassay.

### 2.2. Fourier-Transform Infrared Spectroscopy (FTIR)

FTIR was used to investigate the secondary metabolites, functional groups, and structural features of chlorophyllin. The calculated spectra reflect the well-known dependence of chlorophyllin’s optical properties by measuring infrared intensity against the wavelength of light, as described by Baker et al. [16] and Khan et al. [17].

### 2.3. Singlet Oxygen (^1^O_2_) Quantum Yield Q_Δ_ of Chlorophyllin

One gram of prepared chlorophyllin was dissolved in distilled water. The specimen was subjected to a Shimadzu UV-1900 UV-VIS Spectrophotometer (Livingston, UK) to collect the steady-state absorption spectra, and a Hamamatsu H10330-45 NIR detector was used in the emission mode to collect singlet oxygen (^1^O_2_) decay at 1270 nm at the Chemistry Lab in the Faculty of Science, Ain Shams University, Cairo, Egypt, as previously described [18,19]. The excitation source was a Q-smart Nd: YAG Quantel Laser (Lumibird, Lannio, France) at 355 nm. The singlet oxygen quantum yield was determined by comparing the luminescence intensity of singlet oxygen at 1270 nm photosensitized by chlorophyllin in aqueous with that obtained from the reference phenalenone, which is a universal reference compound with value = 1.0 for the determination of quantum yields *Q*_Δ_ of singlet oxygen, with a remarkable capacity to transfer energy from its triplet excited state to the ground-state molecular oxygen, to finally produce ^1^O_2_ in a process called photosensitization, with very efficient sensitization [20].

### 2.4. Mosquito Rearing

Field-collected larvae were used to raise a laboratory colony of *Aedes aegypti*, and then the females were allowed a blood meal from a pigeon. The second generation was selected for the experiments. All of the experiments were conducted under controlled laboratory conditions at 27 ± 2 °C, 70 ± 5% RH, and a 14:10 h light/dark photoperiod [21].

### 2.5. Laboratory Evaluation of Used Bio-Pesticides

Bioassays were conducted to evaluate the toxicity of chlorophyllin, pheophorbide, and *Bacillus sphaericus* against third-instar *Aedes aegypti* larvae. Different concentrations of each larvicide (chlorophyllin: 0.1, 0.5, 1, 2, 3 ppm; pheophorbide: 1, 2, 3, 4, 5 ppm; *Bacillus sphaericus*: 1, 2, 3, 4, 5 ppm) were prepared. Glass beakers with 100 mL of distilled water received 20 third-instar larvae of *Aedes aegypti* and one milliliter of the tested substance. Distilled water alone was utilized as a control. Three replications of each treatment and control were made. Chlorophyllin was incubated in the dark for about 18 h and then exposed to sunlight.

Larval mortality was recorded 24 h post-exposure based on loss of any movement, even after a mild touch with a glass rod [22,23]. The mortality rate was corrected and plotted against concentrations. LC_50_ and LC_90_ values were estimated for each larvicide and used for the field applications.

### 2.6. Field Evaluation of Used Pesticides

Semi-field experiments were applied in three different sites in the Hurghada region, Red Sea Governorate, Egypt, which were previously examined as positive sites for *Aedes aegypti* breeding [3]. One liter of unfiltered water from town cesspits serving as *Aedes aegypti* breeding sites was introduced in plastic containers and implanted in the same breeding places to be exposed to the same environmental conditions. The cesspit water contained suspended matter, organic compounds, and debris. Fifty larvae per liter of cesspit water were introduced into each plastic bowl at 10 p.m. in full darkness.

Guided by the laboratory LC_90_ values, different concentrations of each tested bio-insecticide (chlorophyllin: 10, 50, 100, 200, 300 ppm; pheophorbide: 20, 50, 100, 200, 300 ppm; *Bacillus sphaericus*: 200, 300, 400, 500, 600 ppm) were prepared and applied at a volume of one milliliter to 100 mL of water in each bowl, and they were then exposed to natural sunlight from sunrise to sunset. Control experiments were performed in the absence of chlorophyllin, pheophorbide, and *Bs*. Mortality readings were recorded after 24 hr., corrected, and used to calculate field values of the LC_50_ and LC_90_, toxicity index, and relative potency.

### 2.7. Calculating the Toxicity Index and Relative Potency

Toxicity index formula [24]:

     LC_50_ of the most toxic insecticide Toxicity index = ---------------------------------------------------------- × 100     LC_50_ of (less toxic) tested insecticides used

     LC_50_ for least used insecticide competence (Lowest toxic) Relative potency = ----------------------------------------------------------------      LC_50_ for other insecticides tested

### 2.8. Genotoxicity Studies

The effect of chlorophyllin application (compared with pheophorbide and *Bs*) on the DNA configuration of treated *Aedes aegypti* 3rd-instar larvae after 24 h of exposure was studied using the Random Amplified Polymorphic DNA (RAPD) PCR technique. DNA extraction was performed using the Dneasy Mini Kit (QIAGEN, Taufkirchen, Germany). DNA was extracted as described by Nieman et al. [25], while the PCR cycles were performed as described by Williams et al. [26]. The primers used during amplification, along with their sequences, are tabulated in Table 1. List of the primer sequences used for RAPD-PCR). The similarity index quantifies the degree of band sharing between individuals and is calculated using the following formula:

$$\mathrm{Similarity}\;\mathrm{Index}=\frac{2N\;ab}{Na+Nb}$$

where *N ab* represents the number of bands common to individuals *a* and *b*, while *Na* and *Nb* denote the total number of bands present in individuals *a* and *b*, respectively [27].

### 2.9. Histopathological Changes

Histopathological examinations of chlorophyllin-treated third-instar mosquito larvae after 24 h of exposure were conducted following the methodologies described by Farida et al. [28] as routine preparations of microscopic slides to assess tissue alterations.

### 2.10. Statistical Analysis

Based on the control treatments, Abbott’s formula [29] was used to obtain the corrected mortality percentages. Using Analyst Soft Biostat Pro V 5.8.4.3 Software, the dose–response relationship curve was statistically estimated by probit analysis [30] to determine each insecticide toxicity line’s regression equation components and lethal concentrations.

## 3. Results

### 3.1. Chlorophyllin Characterization (FTIR Analysis)

**Figure 1:**
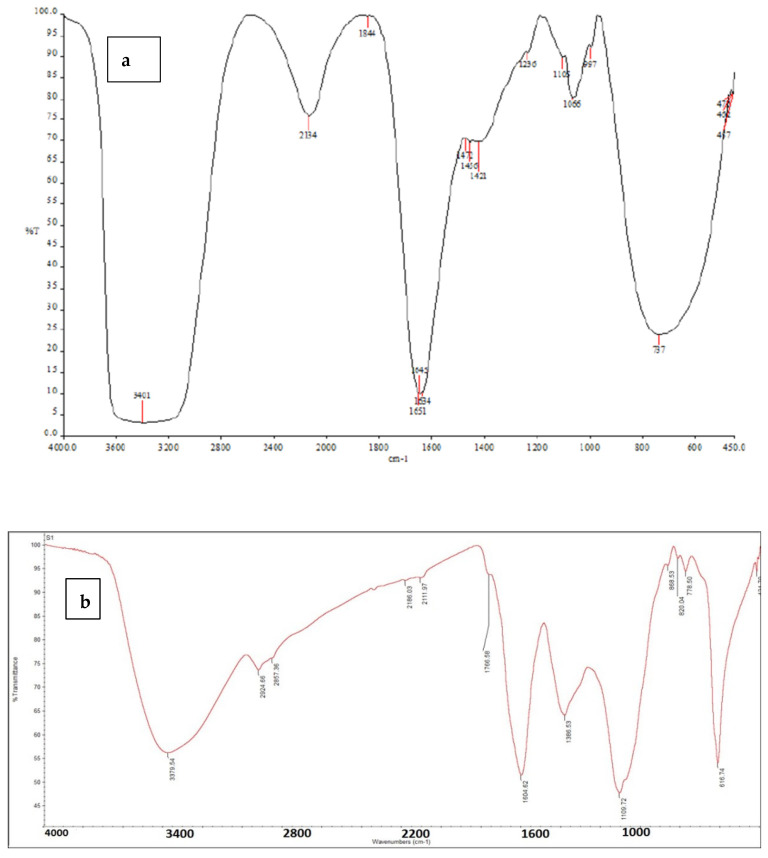
FTIR spectra: (**a**) Standard IR spectrum of chlorophyllin (research gate). (**b**) The FTIR spectrum of the Na-Cu-chlorophyllin complex.

The FTIR technique revealed a broad band of N-H and O-H functional groups at 3379 cm^−1^. The presence of the sp^3^ C-H bond is indicated by the peaks at 2924 cm^−1^ and 2857 cm^−1^. Asymmetric carboxylate stretching and the wide absorption band indicate C=N at 1604 cm^−1^. Furthermore, a symmetric carboxylate group is present in the band at 1386 cm^−1^. The 1110 cm^−1^ and 779 cm^−1^ bands correspond to the chlorophyllin’s C-O stretching and C-C functional groups, respectively. The Cu-N bond stretching is responsible for the strong band at 617 cm^−1^ (Figure 1a,b).

### 3.2. Singlet Oxygen Quantum Yield of Chlorophyllin

**Figure 2:**
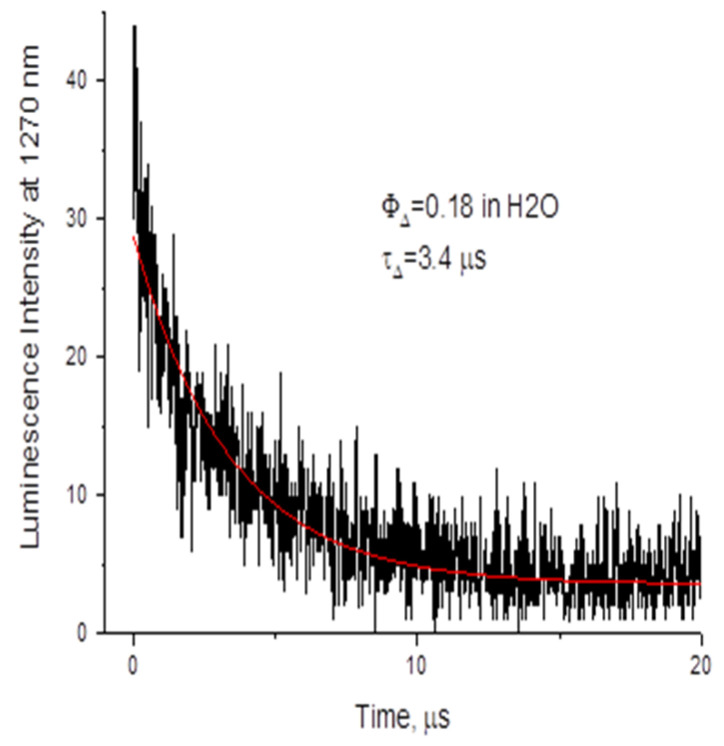
Singlet oxygen decay at 1270 nm when photosensitized by chlorophyllin in H_2_O solution; Φ: quantum yield value; τ: singlet oxygen lifetime.

The singlet oxygen quantum yield of chlorophyllin is greater than zero, as determined by comparing its luminescence intensity (at 1270 nm) against the reference compound phenalenone. The singlet oxygen quantum yield obtained from chlorophyllin was recorded as 0.18 compared to phenalenone as a reference, recording one unit and a singlet oxygen lifetime of 3.4 µs, as shown in Figure 2.

### 3.3. Toxicity of Tested Bio-Pesticides

The mortality rate, LC_50_ and LC_90_, toxicity indices, and relative potencies are presented in Table 2. Laboratory evaluation of Na-Cu chlorophyllin, pheophorbide, and Bacillus *sphaericus* against Aedes *aegypti* larvae after 24 h of exposure) and Table 3. Semi-field evaluation of Na-Cu chlorophyllin, pheophorbide, and Bacillus *sphaericus* against Aedes *aegypti larvae* after 24 h of exposure. The probit analysis presented in Table 2. Laboratory evaluation of Na-Cu chlorophyllin, pheophorbide, and *Bacillus sphaericus* against *Aedes aegypti* larvae after 24 h of exposure demonstrates the toxicity of Na-Cu chlorophyllin, pheophorbide, and *Bs* against *Aedes aegypti* larvae under laboratory conditions, with LC_50_ values of 0.47 ± (0.37–0.60), 1.87 ± (1.60–2.12), and 2.07 ± (1.80–2.30) ppm, respectively. In the semi-field application, as shown in Table 3. Semi-field evaluation of Na-Cu chlorophyllin, pheophorbide, and *Bacillus sphaericus* against *Aedes aegypti* larvae after 24 h of exposure the comparative toxicity further confirmed Na-Cu chlorophyllin as the most potent larvicide, with an LC_50_ value of 93.3 ± (75.90–114.56). This consistent potency across both controlled and semi-field conditions underscores the effectiveness of Na-Cu chlorophyllin as a bio-pesticide against *Aedes aegypti* larvae.

### 3.4. Genotoxicity Studies

**Figure 3:**
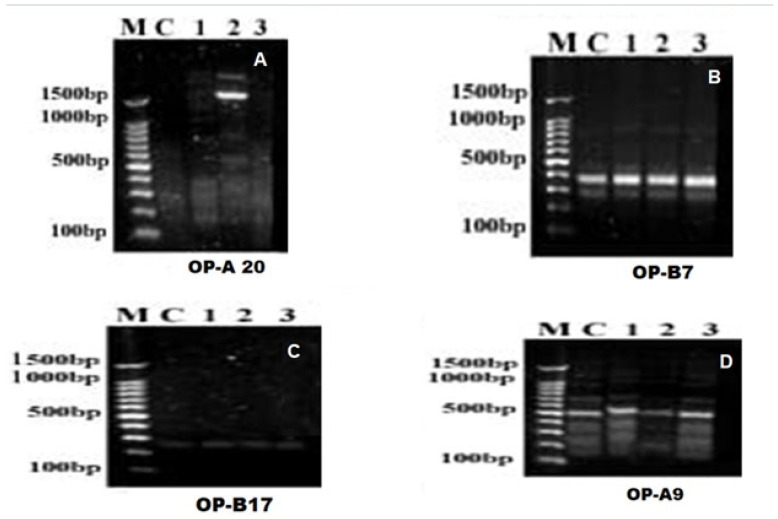
RAPD-PCR was produced for *Aedes aegypti* 3^rd^-instar larvae using the following primers: (**A**) OP-A20, (**B**) OP-B7, (**C**) OP-B17, and (**D**) OP-A9.

The RAPD-PCR results for the treated *Aedes* larvae after 24 h of exposure were analyzed using different primers (OP-A20, OP-B 7, OP-B 17, and OP-A20) to track the deviation of the genomic DNA of the *Aedes* larvae after treatment with chlorophyllin, pheophorbide, and *Bs*, compared with the DNA of healthy, non-treated larvae, as shown in Table 4, Table 5, Table 6, and Table 7, respectively. As shown in Figure 3, the bands generated by primers across these treatments were subjected to a similarity index calculation to assess the genetic relationships.

The RAPD-PCR results proved great similarities in DNA configuration after the treatment of *Aedes* larvae with chlorophyllin compared with normal DNA. Treatment with pheophorbide had an obvious effect on larval DNA after treatment, while treatment with *Bacillus sphericus* proved to be genotoxic, with a great deviation compared with chlorophyllin.

### 3.5. Histopathological Changes

**Figure 4:**
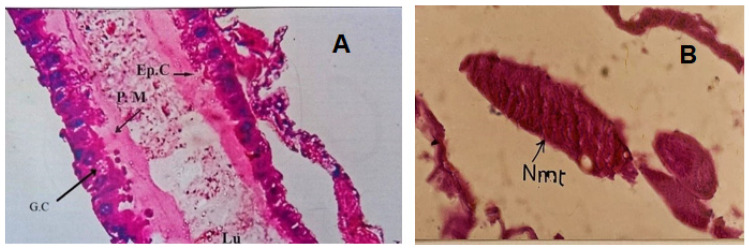
Longitudinal section (L.S.) of normal untreated *Aedes aegypti* larva (×100) after 24 h of exposure, showing (**A**) normal gut epithelial cells (Ep.C), peritrophic membrane (P.M), gastric caeca (G.C), and lumen (Lu); (**B**) normal muscles (Nm).

**Figure 5:**
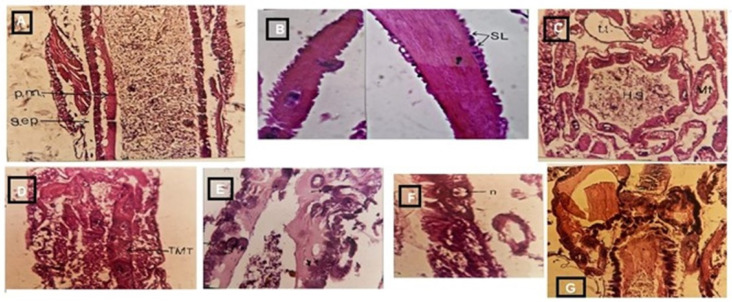
L.S. in *A. aegypti* larva (×100) treated with chlorophyllin after 24 h of exposure, showing (**A**) disruption of gut epithelial cells and peritrophic membrane; (**B**) disruption of myofibrils in muscles; (**C**) epithelial cells of the hindgut, Malpighian tubules, and muscles; (**D**) treated Malpighian tubules (TMTs); (**E**) irregular shape of epithelial cells; (**F**) midgut epithelia with an empty apical zone from the cytoplasm, exfoliation of the brush border, and empty nucleus; (**G**) disruption of epithelial cells with empty apical zone. gep: gut epithelial cells, p.m: peritrophic membrane, SL: sarcolemma, M: muscle, Mt: Malpighian tubule, n: nucleus.

After the treatment of *Aedes aegypti* larvae with 24 h of exposure to chlorophyllin, the following histological changes were recorded compared to the control (Figure 4): treated epithelial cells of the midgut protrusion, with the brush border completely disordered and thinning out. The epithelial cells lost their regular shape, detached from each other, changed their affinity for the dye, and became darker. Shrinkage of the cytoplasm was noticeable, and the irregular shape of gut cells was also seen. The cytoplasm appeared dense in some parts but empty and irregular in others, and the elongation of epithelial cells was clear. The light and dark bands in the treated muscles could not be detected, and the sarcolemma lost its continuity and appeared disrupted. After larval treatment with chlorophyllin, the Malpighian tubules lost their crescent shape and appeared with irregular nuclei (Figure 5).

## 4. Discussion

The re-emergence of *Aedes aegypti* within the Red Sea region in Egypt [3] encouraged us to apply photosensitizers against *Aedes aegypti* larvae as vector of dengue fever in Egypt. Chlorophyllin is a photosensitizer and a water-soluble analog of the famous green pigment chlorophyll [31]. Pheophorbide is an intermediate product in the chlorophyll degradation pathway [32].

This study employed a multi-faceted approach to investigate chlorophyllin’s potential efficiency as a novel control agent against the larvae of the dengue vector *Aedes aegypti*. First, we characterized the key properties of chlorophyllin, including its singlet oxygen quantum yield, and evaluated its larvicidal efficacy under controlled laboratory and semi-field conditions. Second, we investigated the genotoxic effects of chlorophyllin on *Aedes aegypti* larvae using Random Amplified Polymorphic DNA (RAPD)-PCR. Finally, a histopathological analysis of the treated larvae was conducted to further understand the mechanism of action and identify the pathogenicity.

Our results demonstrated that chlorophyllin (Ch-L) could kill mosquito larvae in a few hours after exposure to sunlight under laboratory and semi-field conditions, with the lowest LC_50_ values compared with pheophorbide or *Bs*. Wohllebe et al. [33] found that chlorophyllin at 8 ng was enough to induce photodynamic damage and death in mosquito larvae. We prepared chlorophyllin in our laboratory from the peels and leaves of vegetables, and then we used it to prepare a Cu-Mg chlorophyllin derivative, which proved its high toxicity. Trials to control mosquitoes using porphyrin, Rose Bengal, and xanthene as photosensitizing agents were previously carried out by Younis et al. [34] and Meier and Hillyer [35]. The larvicidal activity of Ch-L against water-dwelling disease vectors was also proven by Abdel-Kader et al. [8] and Elshemy et al. [36]. Some factors interfere with the photodynamic activity of chlorophyllin, such as the concentration of photosensitizers used, accumulation of chlorophyll derivatives (incubation in the dark), and light exposure period; longer incubation in the dark and more prolonged exposure to light increase larval mortality [9]. We assumed that chlorophyll derivatives need to spread inside the larval body before starting the photodynamic reaction. Abdel Kader and El-Tayeb [8] deduced that chlorophyll products could kill mosquito larvae in the presence of solar radiation after incubation in the dark. Erzinger et al. [37] showed that, in *Chaoborus* sp., a dark incubation period of about 3 h is sufficient to induce mortality of about 90%.

Most cell and tissue components are unsuitable acceptors of electronic energy from ^3^Sens (triplet excited state of photosensitizer), since their triplet states are too energetic. O_2_ represents ground-state oxygen. Energy transfer occurred from the triplet excited state of the photosensitizer to ground-state oxygen, so singlet excited oxygen was formed, whose energy level lies at only 22.5 kcal [38].

^3^Sens + ^3^O_2_ ↔ Sens + ^1^O_2_

Estimating the quantum yield of chlorophyllin is critical to showing its photodynamic properties. By measuring the efficiency of chlorophyllin in transferring energy from the triplet excited state of the photosensitizer to ground-state oxygen, singlet excited oxygen was formed. Chlorophyll’s photodynamic interaction generates reactive oxygen species (ROS) and reactive singlet oxygen that can cause structural disintegration, leading to cell necrosis and apoptosis [39,40].

We used the RAPD-PCR technique to detect any alterations in the treated larvae’s DNA produced by environmental genotoxic stress. This technique was previously used to monitor DNA changes by Gupta and Preet [41]. The genotoxicity results proved that chlorophyllin did not induce DNA alterations or mutations when compared with other mosquitocides, such as pheophorbide. Treatment with *Bs* could alter the DNA configuration in an obvious way. This is one of the important advantages of using chlorophyllin in insect control.

Histopathological signs proved that chlorophyllin is a gut poison that interrupts epithelial cells’ integrity and leads to cell apoptosis. Fat and muscle tissues were also affected. Histopathology proved significant damage to the alimentary canal and its architecture. Some similar pathological evidence was found by [42,43,44], who recorded tissue disruption after the treatment of mosquitoes with different plant extracts.

We concluded that chlorophyllin is a stomach poison and represents a new approach in insect control. Chlorophyllin is a promising mosquito larvicide with many advantages. Derivatives of chlorophyll do not harm vertebrates or humans, for example, as chlorophyllin is an approved food ingredient (E 170) [9,45]. They also do not pose any toxicity to non-transparent bodies. Chlorophyll derivatives can be easily extracted from various plant resources, and this approach appears to have economic value. Chlorophyll derivatives are sufficiently active to cause photodynamic death even in small quantities. Photosensitive chlorophyllin degrades very fast, without the formation of toxic byproducts, making it environmentally sound and economically safe [33].

## 5. Conclusions

We concluded that chlorophyllin is very effective against mosquito larvae. Chlorophyllin can absorb light of a specific wavelength and transform it into valuable energy. Combining chlorophyllin with light and molecular oxygen causes cell death by generating reactive oxygen species (ROS), which cause cell necrosis and apoptosis. It was found that chlorophyllin has no dramatic genetic toxicity, so it is recommended to be used in mosquito control strategies because it is safe and non-mutagenic. Treatment with chlorophyllin appeared to show histopathological signs, especially in the gut region, so we recommend its use as a stomach poison.

## Figures and Tables

**Table 1:** List of the primer sequences used for RAPD-PCR.

Primer Name	Sequence
Operon (OP)-A9	5′-GGGTAACGC-3′
OP-B17	5′-GGTGACGCAG-3′
OP-B7	5′-GGTGACGCAG-3′
OP-A20	5′-GTTGCGATCC-3′

**Table 2:** Laboratory evaluation of Na-Cu chlorophyllin, pheophorbide, and *Bacillus sphaericus* against *Aedes aegypti* larvae after 24 h of exposure.

Tasted Compounds	Concentration (ppm)	Percentage Mortality (%)	LC_50_ (ppm) (Co. Limit)	LC_90_ (ppm) (Co. Limit)	Slope ± SE	χ^2^	*p*	Toxicity Index	Relative Potency
**Chlorophyllin**	0.1	23.33	0.47 (0.37–0.6)	4.88 (3.4–8.1)	1.26 ± 0.12	12.18	0.005	100	4.40
	0.5	46.77							
	1	61.25							
	2	75							
	3	92							
**Pheophorbide**	1	31.77	1.87 (1.6–2.12)	6.8 (5.55–9.28)	2.28 ± 0.25	9.55	0.02	25.11	1.10
	2	46.77							
	3	61.77							
	4	76.77							
	5	90.77							
***Bacillus sphaericus***	1	22.500	2.07 (1.8–2.3)	6.1 (5.2–7.7)	2.7 ± 0.3	3.6	0.3	22.71	1
	2	45							
	3	63.333							
	4	76.666							
	5	90							

**Table 3:** Semi-field evaluation of Na-Cu chlorophyllin, pheophorbide, and *Bacillus sphaericus* against *Aedes aegypti* larvae after 24 h of exposure.

Tasted Compounds	Concentration (ppm)	Percentage Mortality (%)	LC_50_ (ppm) (Co. Limit)	LC_90_ (ppm) (Co. Limit)	Slope ± SE	χ^2^	*p*	Toxicity Index	Relative Potency
**Chlorophyllin**	10	13.33	93.3 (75.9–114.56)	810.07 (545.76–1420.010)	1.36 ± 0.14	12.72	0.059	100	4.31
	50	26.77							
	100	48.33							
	200	73.33							
	300	76.6							
**Pheophorbide**	20	16.77	92.77 (77.49–110.89)	622.35 (440.82–1016.5)	1.5 ± 0.15	1.47	0.68	100.56	4.34
	50	31.77							
	100	51.77							
	200	66.77							
	300	81.77							
***Bacillus sphaericus***	200	13.333	402.41 (376.04–431.6)	830.45 (724.74–1007.74)	4.07 ± 0.39	2.34	0.5	23.19	1
	300	27.500							
	400	46.667							
	500	63.333							
	600	80							

**Table 4:** The total number and size of RAPD-PCR fragments generated by the OP-A20 primer from treated *Aedes* larvae, with LC_50_ values.

Bands	Control	Chlorophyllin	Pheophorbide	*Bs*
**1**	-	-	-	-
**2**	1680	1680	1765	
**3**	-	-	1670	1075
**4**	932	932	932	932
**5**	765	765	765	765
**6**	650	-	-	650
**7**	-	670	670	-
**8**	365	365	-	365
**9**			315	315
**10**	-	-	245	245
**Total**	5	5	7	7
**Similarity Index**	-	0.8	0.5	0.6

**Table 5:** Total number and size of RAPD-PCR fragments generated by the primer OP-B7 in treated *Aedes* larvae, with LC_50_ values.

Bands	Control	Chlorophyllin	Pheophorbide	*Bs*
1	1351	1351	1351	-
2	-	-	1270	1270
3	-	-	-	-
4	630	630	630	630
5	460	460		-
6	375	375	-	375
7	235	235	235	235
Total	5	5	4	4
Similarity Index		1	0.6	0.4

**Table 6:** Total number and size of RAPD-PCR fragments generated by the primer OP-B17 in *Aedes* larvae, with LC_50_ values.

Bands	Control	Chlorophyllin	Pheophorbide	*Bs*
1	-	-	-	
2	-	-	-	1477
3	-	-	-	
4	-	-	-	945
5	484	484	520	484
6	300	300	300	300
Total	2	2	2	4
Similarity Index		1	1	0.6

**Table 7:** Total number and size of RAPD-PCR fragments generated by the primer OP-A9 in treated *Aedes* larvae, with LC_50_ values.

Bands	Control	Chlorophyllin	Pheophorbide	*Bs*
1	-	-	-	1535
2	1250	1250	-	-
3	-	-	-	1270
4	-	-	1020	1080
5	811	811	811	825
6	-	-	-	785
7	610	610	620	
8	400	400	400	400
9	300	300	300	300
Total	5	5	5	7
Similarity Index		1	0.8	0.5

## Data Availability

All obtained data are presented and attached to the manuscript.

## Abbreviations

The following abbreviations are used in this manuscript:

^1^O_2_	Singlet oxygen
Ch-L	Chlorophyllin
ROS	Reactive oxygen species
*Bs*	*Bacillus sphaericus*
